# Nutritional Requirements of Lung Transplant Recipients: Challenges and Considerations

**DOI:** 10.3390/nu10060790

**Published:** 2018-06-19

**Authors:** Valerie Jomphe, Larry C. Lands, Genevieve Mailhot

**Affiliations:** 1 Lung Transplant Program, Centre Hospitalier de l’Université de Montréal, 900 Saint-Denis Street, Montreal, QC H2X 0A9, Canada; valerie.jomphe.chum@ssss.gouv.qc.ca (V.J.); larry.lands@mcgill.ca (L.C.L.); 2 Department of Pediatrics, Montreal Children’s Hospital—McGill University Health Centre, 1001 Décarie Boulevard, Montreal, QC H4A 3J1, Canada; 3 Meakins Christie Laboratories, Research Institute of the McGill University Health Centre, 1001 Décarie Boulevard, Montreal, QC H4A 3J1, Canada; 4 Department of Nutrition, Faculty of Medicine, Université de Montreal, 2405 Cote Sainte-Catherine Rd., Montreal, QC H3T 1A8, Canada; 5 Research Centre, CHU Sainte-Justine, 3175 Cote Sainte-Catherine Rd., Montreal, QC H3T 1C5, Canada

**Keywords:** lung transplantation, nutritional status, nutrient requirements, nutrition management, nutritional recommendations, macronutrients, micronutrients

## Abstract

An optimal nutritional status is associated with better post-transplant outcomes and survival. Post-lung transplant nutrition management is however particularly challenging as lung recipients represent a very heterogeneous group of patients in terms of age, underlying diseases, weight status and presence of comorbidities. Furthermore, the post-transplant period encompasses several stages characterized by physiological and pathophysiological changes that affect nutritional status of patients and necessitate tailored nutrition management. We provide an overview of the current state of knowledge regarding nutritional requirements in the post-lung transplant period from the immediate post-operative phase to long-term follow-up. In the immediate post-transplantation phase, the high doses of immunosuppressants and corticosteroids, the goal of maintaining hemodynamic stability, the presence of a catabolic state, and the wound healing process increase nutritional demands and lead to metabolic perturbations that necessitate nutritional interventions. As time from transplantation increases, complications such as obesity, osteoporosis, cancer, diabetes, and kidney disease, may develop and require adjustments to nutrition management. Until specific nutritional guidelines for lung recipients are elaborated, recommendations regarding nutrient requirements are formulated to provide guidance for clinicians caring for these patients. Finally, the management of recipients with special considerations is also briefly addressed.

## 1. Introduction

Lung transplantation (LTx) is a life-saving treatment modality for selected individuals with irreversible end-stage lung diseases. Indications for LTx fall broadly into four categories: obstructive (e.g., chronic obstructive pulmonary disease (COPD)), suppurative (e.g., cystic fibrosis (CF) and bronchiectasis), restrictive (e.g., interstitial lung disease (ILD), connective tissue disease, sarcoidosis) and vascular (e.g., pulmonary arterial hypertension). From a nutritional standpoint, lung transplant (LTx) recipients represent a unique subpopulation of patients with highly heterogeneous demographic and clinical features. Patients with CF are younger, have a lifelong pancreatic insufficiency causing maldigestion and malabsorption, are more likely to be underweight, and to suffer from pretransplant diabetes. Conversely, individuals with COPD and ILD are older, as those conditions develop progressively over several years, may have a significant history of past smoking and in contrast to CF patients, are more frequently overweight and obese. The wide age range, the clinical heterogeneity of the underlying condition, the presence of distinct pre- and post-transplant comorbidities and the varying body mass index (BMI) status and health lifestyles all influence nutritional requirements of these patients in their own distinct manner. 

The post-LTx period is subdivided into several stages, each of which is characterized by physiological and pathophysiological changes that may have an impact on nutrition requirements. In the early post-operative phase (i.e., from transplant surgery to six months post), surgical wounds increase nutrient requirements necessary for the healing process. In parallel, nutrition should also support new metabolic demands while replenishing depleted energy stores and ensuring graft survival. Concomitantly, infections, surgical and digestive complications, primary graft dysfunction and rejection are potential early to mid-stage complications that may necessitate nutritional adjustments. Over the long term (i.e., >2 years post-transplant), the occurrence of osteoporosis, malignancies, and gastrointestinal, renal, cardiovascular and metabolic diseases may all require specific nutritional intervention. Irrespective of post-transplant stages, lifetime immunosuppressive and corticosteroid therapy has acute and chronic adverse effects that affect nutrition including improved appetite, altered taste, gastrointestinal symptoms, hyperglycemia, hyperlipidemia and electrolyte disturbances [1]. Altogether, individual-, primary disease- and transplant-related factors make nutrition management particularly challenging and the use of a single approach impossible.

Despite this unique combination of peculiarities, little research has been conducted on post-transplant nutritional status and interventions in these patients and no specific nutrition therapy guidelines have yet been developed for this population. The goal of this narrative review is to provide a knowledge base on nutritional status and nutritional requirements of LTx recipients. Focus has been given to the period of time beginning at transplant; therefore pre-transplant nutrition management is not covered but has been the topic of a previous review [2]. 

## 2. Nutritional Status of Lung Transplant Recipients

### 2.1. Energy Metabolism and Weight Regulation

Substantial weight gain and changes in body composition have been consistently reported in both the adult and pediatric settings after LTx [3,4,5,6,7,8]. Weight changes are more dynamic in the early post-transplant period but tend to stabilize as time from transplant increases [9]. While body weight generally increases after LTx, some patients experience weight loss caused unintentionally by the occurrence of post-transplant complications. 

In a cohort of 35 adult recipients, Madill et al. [7] were the first to report a paradoxical weight gain at six and 12 months post-LTx in recipients with CF and bronchiectasis despite a decrease in total calorie intake and persistence of pancreatic insufficiency in the CF patients. The authors explained this finding by the reduction in energy expenditure due to the correction of an underlying hypermetabolic state related to the pre-LTx terminal lung disease. Consistent with this, resting energy expenditure (REE), as assessed by means of indirect calorimetry, decreased from 132 ± 18% prior to transplant (8.3 ± 7.5 months) to 112 ± 16% post-LTx (3.9 ± 3.7 months) with improvements in BMI and weight, but not height z-scores at one and two years post-LTx in pediatric lung recipients, mainly with CF [5]. However, no association was detected between the REE, weight/BMI changes and forced expiratory volume in 1 s (FEV_1_), a finding that was attributed to the small sample size (*n* = 22) [5]. Interestingly, the lowest pre-transplant REE values were measured in non-CF patients, suggesting CF-specific metabolic demands associated with recurrent infections, chronic inflammation and increased respiratory effort. REE measurements have not been yet reported in adult lung recipients and energy requirements are currently estimated using predictive formulas that are of limited accuracy and not specific to the transplant population. Estimated calorie requirements are multiplied by a stress and activity factor appropriate for the patient’s condition and could be further adjusted if weight control is necessary. Since energy metabolism is not static and likely to change throughout the post-transplant course, periodic reassessment of calorie needs should be part of the post-transplant nutrition management as weight gain may have serious health implications for those who are overweight and obese. Indeed, Forli et al. investigated the occurrence of metabolic syndrome among 35 lung recipients followed up for two years [10]. Over this relatively short period of time, five patients developed metabolic syndrome. These patients were more likely to have a lower lean mass and a higher weight gain, BMI, leptin levels, and trunk fat than those who did not develop the syndrome. The authors raised the possibility that lung recipients exhibited abnormalities of energy homeostasis after LTx. Indeed, lung recipients experienced a significant increase in leptin levels, a satiety hormone produced by adipose tissue and known to reduce food intake and increase energy expenditure in order to avoid excessive body fat storage. As expected, energy intake decreased after transplantation; however weight and body fat did not stabilize and kept increasing, which is opposite to what is expected in terms of homeostatic responses. It was speculated that lung recipients exhibited a degree of leptin resistance that did not affect the control of food intake but alter other pathways involved in the regulation of body composition. One cause for such leptin resistance may be the intake of corticosteroids, which were shown to antagonize leptin action by attenuating leptin receptor signal transduction and decreasing central leptin sensitivity [11]. It is unlikely that the increased amount of body fat is the sole contributor to the increase in serum leptin as adiponectin levels, another fat-derived hormone, remained unchanged after LTx.

Since corticosteroids are known to decrease fat oxidation, promote protein catabolism and reduce protein synthesis [12,13], it is generally assumed that post-transplant weight gain is primarily due to a gain in fat mass. Kyle et al. [14] challenged this assumption by showing that post-LTx weight gain was in fact the result of a concomitant increase in fat-free mass and body fat in adult recipients. Using bioimpedance measurements, they demonstrated dynamic changes within these two compartments with an initial decline in the early post-operative period likely ascribed to a combination of insufficient intakes, limited physical activity and high metabolic demands, followed by a progressive recovery that exceeded pre-transplant values by six to nine months post-transplant [14]. As a result, nearly two-thirds of patients reached normal and even high fat-free mass values by year 2. Interestingly, they compared two eras characterized by a significant change in corticosteroid doses and found similar weight gains between those who were given the lower vs. higher dose; however the former group experienced a greater gain in fat-free mass suggesting that the impact of corticosteroids on protein and fat metabolism depends on the dose used. In a longitudinal study of pediatric patients, body composition was measured prior to transplant and at one and two years post-transplant by means of dual energy X-ray absorptiometry (DXA). A significant increase in height, weight, and BMI was noted at one and two years, although values remained below the 50th percentile for age, a finding attributed to enteral feeding discontinuation, suboptimal food intakes and increase in metabolism due to allograft rejection and infections. Cumulative growth deficits in addition to requirements to sustain growth velocity may also explain the failure to catch up with age-based norms within such a short period of time [6]. In contrast to adult recipients where a gain in fat-free mass and body fat was reported [10,14], pediatric recipients gained primarily lean mass, a finding that may reflect the lower dose of corticosteroids used in this population, age of participants as aging is associated with a preferential loss of lean mass and gain in fat mass, and the relative homogeneity of the study population, made up of a majority of CF patients. 

Besides immunosuppressive therapy, other factors were shown to influence post-transplant weight changes, of which BMI status prior to or at transplant is considered a strong predictor. Underweight patients gained on average more weight than normal-weight, overweight and obese recipients [3]. Since underweight patients are more likely to have CF while COPD and ILD patients are overrepresented in overweight and obese categories, it remains difficult, in some cases, to discriminate the impact of the clearance of the underlying respiratory disease from that of the BMI on post-transplant weight changes. 

With the exception of survival data, clinical implications of post-transplant weight changes have not been extensively studied due to the limited survival of lung recipients, which is an inherent barrier to the conduct of prospective longitudinal studies over a long period of time. In one of the few longitudinal studies spanning up to four years post-transplant, patients diagnosed with obliterative bronchiolitis consistently showed loss of body weight, fat-free mass, and body fat [14]. Whether such changes precede the onset of or occur early in the course of bronchiolitis obliterans syndrome (BOS) and contribute to the poor prognosis need to be clarified. Conversely, a higher weight gain in the first year post-transplant was associated with reduced subsequent mortality [8]. Again, it remains unclear whether early weight gain results from or leads to a better allograft function. Assuming that weight gain is a side effect to immunosuppressive therapy, one may argue that post-transplant weight gain may also reflect an increased efficacy to immunosuppressive drugs. Singer et al. documented that death from an infectious cause beyond the first year post-transplant was more prevalent among patients who gained more weight whereas the number of deaths due to a malignancy was greater in those who gained less weight, however the presence of an undiagnosed cancer may have contributed to limit weight gain [8]. Likewise, Madill et al. found that sepsis was the major cause of early death in recipients with BMI < 25 kg/m^2^ while in patients with BMI > 25, causes of death were more diverse and less often attributed to infections [15]. They postulated that the combination of *Burkholderia cepacia* positivity and low BMI, often seen in CF patients, resulted in increased mortality based on the premise that adequately nourished individuals are more able to fight infections. In the largest cohort examined thus far, Allen et al. found decreased survival among underweight, overweight and obese recipients compared to normal-weight patients [16]. Interestingly, after censoring deaths in the year following transplant, survival differences between BMI categories were no longer significant, suggesting that such differences were likely driven by events occurring in the first year after transplant, an observation supported by Lederer et al., who showed that obesity was an independent risk factor for primary lung graft dysfunction [17]. While the link between high pre-transplant BMI and post-transplant survival appears well evidenced [3,18], this association is much less robust among underweight recipients with reports of no effects [19,20], trends towards increased mortality [15,21] and significant impact on mortality [16,22,23,24]. Of note, the association between low BMI and increased mortality was mainly seen in the largest cohort studies [16,22,24] where it was sometimes restricted to specific subgroups such as underweight recipients with COPD [22] or manifested not until 1 year after transplant [16], thereby suggesting different timing and mechanisms for increased mortality in underweight vs. overweight/obese patients.

### 2.2. Macronutrients 

#### 2.2.1. Proteins

Very little research has been conducted regarding protein requirements in lung recipients. Only one study has measured nitrogen balance in 18 lung recipients who stayed in the intensive care unit (ICU) more than four days post-operatively [25]. They found that nitrogen balance, as assessed by the difference between nitrogen intake and output of urea nitrogen, became progressively negative to reach a maximum after five days. Length of ICU stay and immunosuppression were negatively correlated with nitrogen balance while increased protein intake demonstrated a positive association, as expected. However, available data are insufficient to delineate protein requirements of lung recipients at different times post-transplant and hence, no specific recommendations have been made. Only one observational study, describing their experiences with early post-operative nutrition management of 52 lung recipients receiving parenteral nutrition, set initial protein goals between 0.6 and 1.9 g/kg/day with a mean of 1.37 ± 0.25 g/kg [26]. Nearly two-thirds of these patients reached protein goals by day 2 of parenteral nutrition while nine patients failed to reach the desired goals and six exceeded the estimated needs. Overall, these observations indicate that protein requirements are extremely variable in the post-operative period and should be adjusted on an individual basis. Thus far, no studies have been conducted on protein requirements of lung recipients at later post-transplant stages.

#### 2.2.2. Carbohydrates

Hyperglycemia, without preexisting diabetes, is a common metabolic disorder occurring in the early post-transplant period [26]. Causes include physiologic stress related to the surgery, which enhances secretion of epinephrine and cortisol, two hormones acting synergistically to increase hepatic output of glucose and decrease peripheral glucose uptake, insulin secretion, and insulin sensitivity. Calcineurin inhibitors and corticosteroids are also important contributing factors that negatively affect glucose metabolism, and insulin secretion and action. New onset diabetes after transplantation (NODAT) is a comorbidity affecting 40% of patients at two years that is associated with increased mortality [27,28,29]. NODAT incidence is maximal at three months due to a combination of insulin resistance and β-cell dysfunction caused by near-maximal immunosuppression and the presence of the “systemic inflammatory response syndrome”, a proinflammatory status resulting from surgical trauma and infection, that is universally present in the early post-transplant period [28]. NODAT is an irreversible condition in the majority of patients but was shown to resolve in 14% of recipients who survived more than 2 years due to β-cell recovery [28]. Multivariate analysis of risk factors for developing NODAT revealed that CF (HR: 3.3 (CI 95% 2.58–4.21)), recipient BMI ≥ 30 (HR: 1.51 (CI 95% 1.24–1.84)) and tacrolimus intake at hospital discharge (HR: 1.67 (CI 95% 1.39–2.02)) were the variables associated with the greatest risk [30]. Recipient BMI being a modifiable risk factor, prospective studies aimed to determine whether modifying this factor, through nutritional interventions, would influence the development of NODAT are warranted.

#### 2.2.3. Lipids

Fluctuations in serum lipids are commonly observed after LTx and depend upon both disease- and transplant-related factors. While CF lung recipients experienced a rise in total cholesterol, LDL-cholesterol and triglycerides levels after transplant [31], only triglycerides increased significantly in recipients with other lung diseases such as COPD, idiopathic pulmonary fibrosis and sarcoidosis [32]. Differences in median follow-up times (28 months for CF vs. 268 days for COPD and 244 days for idiopathic pulmonary fibrosis and sarcoidosis) and a greater use of statins in non-CF recipients may explain these differences. The fact that a rise in triglycerides levels is a common occurrence in individuals with distinct underlying diseases and at different follow-up times suggests that it is likely a consequence of the immunosuppressant medications. Lipid profile of COPD patients exhibits a distinctive feature with regard to HDL-cholesterol levels, which decreased significantly after transplant, while remaining relatively unchanged in the other diagnoses [31,32]. COPD patients have increased HDL-cholesterol levels prior to transplantation and it is postulated that the fall in serum HDL-cholesterol occurring after transplant is attributed to the removal of a COPD-specific factor produced by the diseased lungs, which exerts HDL-raising effects [32].

In one of the few nutritional intervention studies performed in LTx recipients, Entwistle et al. compared the acceptability and adherence to two dietary interventions, the Mediterranean diet and the Low-Fat Diet, in individuals who had undergone either a lung or heart transplant [33]. Changes in weight and triglycerides after one year of intervention were used to objectively assess adherence and data from all recipients were analyzed together regardless of the organ transplanted. The Mediterranean diet led to a 1.8 kg and a 0.17 mmol/L reduction in weight and triglycerides levels respectively while the Low-Fat Diet resulted in a modest weight loss of 0.2 kg but a greater decline in serum triglycerides (mean of 0.44 mmol/L) [33]. Despite the feasibility nature of this trial, these findings underscore the potential of tailored dietary interventions to partially offset the rise in triglycerides levels observed after transplant. 

### 2.3. Vitamins and Minerals 

No studies have thoroughly looked at water-soluble vitamins (e.g., B-complex vitamins and vitamin C) and minerals (e.g., iron and zinc) and their implications in the context of post-LTx management despite their known involvement in the healing process (e.g., vitamin C, zinc) and energy metabolism (e.g., some B vitamins, iron). The only report of B-vitamin and iron status among lung recipients was done in 26 lung recipients who had a blood workup to evaluate the presence of anemia at a median follow-up of 13.5 months post-LTx [34]. While two-thirds of patients were diagnosed with anemia, iron deficiency was detected only in 35% of patients, folate levels were in the normal range for all subjects and vitamin B12 levels were found to be low in only one participant [34]. These observations suggest that the prevalent anemia observed in lung recipients is primarily of non-nutritional origin [35].

Conversely, fat-soluble vitamins have generated more interest given that the vast majority of CF individuals who undergo LTx display exocrine pancreatic insufficiency and the few pancreatic-sufficient patients who are transplanted develop it over time [36]. The resulting loss of pancreatic enzymes leads to maldigestion and malabsorption of nutrients, especially of fat and fat-soluble vitamins. Reports on the nutritional status of fat-soluble vitamins have therefore largely focused on CF lung recipients, with some exceptions. 

#### 2.3.1. Vitamin D 

Vitamin D refers to two main vitamers: ergocalciferol (i.e., vitamin D_2_), a plant-derived form found in fungi, yeast and some plant-based beverages such as soy milk, that is produced following ultraviolet (UV) irradiation of its precursor ergosterol and cholecalciferol (i.e., vitamin D_3_), naturally occurring in or added to animal-based foods and synthesized in the skin upon UV-B irradiation. The main function of vitamin D is to promote intestinal absorption and renal reabsorption of calcium and phosphorus in order to maintain normal blood levels of these minerals and ensure their sufficient supply for bone mineralization. A growing body of evidence is emerging in support of non-classic roles for vitamin D that extends beyond its actions on calcium and phosphate homeostasis. Among other roles, vitamin D was shown to be involved in the regulation of lung function and immune responses, thereby underscoring its biological relevance in the context of LTx [37]. Indeed, Lowery et al. reported that lung recipients who remained persistently vitamin D-insufficient (i.e., defined as serum 25-hydroxyvitamin D (25OHD) <75 nmol/L (<30 ng/mL)) from transplant up to 1-year post-transplant experienced more bacterial, fungal, cytomegalovirus (CMV) and non-tuberculosis mycobacterial infections and poorer survival than vitamin D-sufficient recipients [38]. In addition, episodes of acute cellular, but not chronic antibody-mediated, rejections were more frequent in the presence of vitamin D insufficiency with a rejection rate that was more than two times that of the non-insufficient group [38]. In contrast, Verleden et al. did not find any association between post-transplant 25OHD levels and the number of infections but reported a positive association between FEV_1_ and 25OHD levels as well as more recipients experiencing moderate to severe B-grade rejections in the vitamin D-insufficient than -sufficient group [39]. Such observations are explained by the ability of vitamin D to act upon both innate and adaptive immune responses through enhancement of the expression of antimicrobial peptides and regulation of dendritic cells maturation and their capacity to induce T-cells with regulatory properties rather than alloreactive potential [40,41]. Although biologically plausible, such mechanisms derive primarily from in vitro and preclinical evidence and have not been directly validated in the context of LTx with the exception of one study performed in the rat allograft LTx whereby the intraperitonally administration of calcitriol (i.e., 1,25(OH)_2_D_3_, the vitamin D bioactive compound) along with a low calcium diet resulted in milder acute rejection and improved lung function compared to control rats fed with the low calcium or a normal diet in the absence of calcitriol [42]. Furthermore, reverse causality cannot be excluded as serum 25OHD, the most reliable marker of vitamin D status, was shown to be sensitive to the presence of inflammation and dropped abruptly after an inflammatory insult, albeit this association was mainly reported during acute-phase responses [43,44]. In the absence of data on inflammatory markers, one might speculate that post-transplant infections and the accompanying inflammation may have in fact contributed to the persistent low-serum 25OHD and the high prevalence of vitamin D deficiency/insufficiency reported in these individuals [38,39,45]. This assumption is further substantiated by the absence of significant clinical improvements in non-CF lung recipients supplemented with 100,000 IU of vitamin D_3_ once monthly for 2 years vs. standard therapy consisting of daily supplements of 880 IU of vitamin D_3_ [46]. Despite the fact that the monthly regimen increased 25OHD levels above the cut-off point of sufficiency associated with optimal immune and respiratory effects in nearly all participants, the authors failed to demonstrate any impact on the occurrence of chronic lung allograft dysfunction as well as acute rejection, lung function, lymphocytic brochiolitis and infection, bone mineral density (BMD), pulmonary and systemic inflammation, and overall survival over the study period [46]. The authors hypothesized that the high prevalence of azithromycin use in their population may have masked the benefits of vitamin D and explained the lack of positive findings. 

#### 2.3.2. Vitamin A and E 

Vitamin A is a generic term that includes both the preformed retinol found in meats and dairy products as well as provitamin A carotenoids (i.e., β-, α-, and γ-carotene, and β-cryptoxanthin) found mostly in plant-based foods. Metabolism of these compounds within the body yields retinoic acid, the bioactive form of vitamin A, which binds to nuclear receptors and modulates transcription of vitamin A-responsive genes involved in cell differentiation, embryogenesis and immune functions. The term vitamin E encompasses a group of eight structurally related compounds, called tocopherols and tocotrienols. Due to its lipophilic nature, vitamin E accumulates in lipid fraction of cellular membranes where it acts as a scavenger of peroxyl radicals and prevents membrane lipid peroxidation and loss of membrane integrity. More recently, other biological roles that go beyond its classical role as an antioxidant have been proposed. Specifically, it is postulated that vitamin E regulates signaling pathways and expression of genes involved in inflammatory, proliferative, metabolic, and antioxidant processes [47,48].

It has been reported that serum levels of vitamin A and E increased after LTx, regardless of the condition causing lung failure and timing of measurements [49]. In CF patients, mean pre-transplant serum vitamin A and E rose from 1.4 ± 0.5 µmol/L and 26.8 ± 8.9 µmol/L to 3.1 ± 1.1 µmol/L and 44.2 ± 16.9 µmol/L respectively after transplantation. High levels were found as early as 6 months post-transplant and remained elevated after more than 4.5 years of follow-up despite no increase in doses of oral vitamin supplementation, and even discontinuation or reduction of supplementation in some patients [49,50]. Current evidence suggests that such alterations are not disease-related but rather caused by the immunosuppressive therapy, which may affect target organs such as the gut, the liver and the kidneys, involved in the absorption, metabolism, storage and clearance of these vitamins. As serum creatinine levels increase and estimated glomerular filtration rate (eGFR) decreases significantly after transplantation, mild renal impairment has been speculated to partly contribute to the rise in serum retinol [49,50]. Liver metabolism of immunosuppressants by cytochrome P450 enzymes may result in functional perturbations that are not detectable by routine liver blood work. Such perturbations may decrease the catabolism of retinol and/or increase the production of the retinol-binding protein and release of retinol from hepatic stores. The rise in serum vitamin E levels is much more difficult to ascribe to factors specifically impacting its homeostasis. It is unknown whether immunosuppressants affect adipose tissue, one of the storage sites of vitamin E, whether they impact the major excretory route of vitamin E via bile or whether they influence the activity of the alpha-tocopherol transfer protein, which facilitates secretion of alpha-tocopherol from liver into the bloodstream [51]. Since vitamin E is exclusively transported by lipoproteins, serum levels of vitamin E are highly influenced by serum lipids, especially cholesterol, and hypercholesterolemia was found to be common in LTx recipients [31,52]. The post-transplant rise in circulating vitamin E may therefore reflect high serum cholesterol. Clinically, increased serum levels of vitamin A and E may have adverse health implications for the LTx recipients, although no confirmatory evidence yet supports this assumption. Particularly relevant to LTx recipients is the inverse relationship between serum vitamin A levels and fracture risk reported in healthy adult males where subjects with serum retinol in the highest quintile displayed a 1.6- and 2.5-fold higher risk of any fracture and hip fracture respectively [53]. In view of the large prevalence of osteopenia and osteoporosis reported in the transplant population, the association between serum retinol and bone health certainly warrants clarification as vitamin A toxicity may further deteriorate the skeleton, already weakened by the immunosuppressive therapy. Hypervitaminosis A is also associated with signs of liver abnormalities, such as elevated transaminases and cirrhosis in the general population. Liver disease is a well-known comorbidity in CF patients and any factor that may contribute to liver deterioration is a concern for this subpopulation of recipients. Vitamin E toxicity is associated with an increased risk of haemorrhage, an adverse effect mainly attributed to its antagonizing action on vitamin K, which is involved in blood clotting [54]. Since vitamin K deficiency is not uncommon in CF patients and may increase the risk of bleeding, consequences of hypervitaminosis E, particularly in recipients with CF, should be addressed. These potential adverse effects nevertheless call for a clinical surveillance of post-transplant vitamin A and E levels and a monitoring and adjustment of oral intakes if deemed necessary.

#### 2.3.3. Vitamin K

Vitamin K is a co-factor of the enzyme gamma-glutamyl-carboxylase, which is responsible for the post-translational carboxylation of glutamic acid residues into gamma-carboxyglutamic acid. This biochemical modification activates vitamin K-dependent proteins such as osteocalcin, matrix-gla protein (MGP), and several anti- and pro-coagulation factors and confers to this vitamin a predominant role in blood coagulation as well as in bone and vascular health. Only one study has reported post-transplant vitamin K status in a small number of CF recipients [55]. Suboptimal vitamin K status, as assessed by the measurement of circulating protein indicated in vitamin K absence (PIVKA-II), was found in 55% of recipients. Although the authors mentioned that the participants received routine vitamin K supplementation, the lack of details regarding the type and dosage of supplement provided, the proportion of patients who took these supplements and their compliance as well as dietary intakes makes such findings difficult to explain and interpret. However, it is clear that the amount of supplemental vitamin K used in this study was insufficient to maintain an adequate status. Contributing factors remain unclear and it was postulated that it might relate to the ongoing pancreatic insufficiency and malabsorption refractory to pancreatic enzyme supplementation. 

Given the potential involvement of vitamin K in bone, the effect of vitamin K_2_, more specifically menaquinone-7, on bone mass was tested in a randomized double-blind controlled trial with 35 lung and 59 heart recipients [56]. Vitamin K supplementation (180 µg/day) was initiated between one and three weeks post-operatively and given for a period of one year. Vitamin K supplementation led to a significant increase in lumbar spine bone mineral content (BMC) of lung, but not heart, recipients while the greatest effect on lumbar spine BMD was observed in heart patients. Reasons for such distinctive effects on BMC and BMD were unclear and may relate to subtle differences in bone turnover between heart and lung patients. However, patients who received vitamin K supplementation also demonstrated an increase in the pro-resorptive parathyroid hormone (PTH) despite no significant changes in serum 25OHD levels, which may have mitigated the effect of vitamin K on bone. Lower compliance rates among those who received the vitamin K (i.e., 82% and 75% in lung and heart recipients respectively) vs. placebo (i.e., 93% and 88%) may have as well influenced the results.

#### 2.3.4. Antioxidants

While a few studies have reported levels of antioxidant vitamins in lung recipients [49,50], only one study has specifically addressed the antioxidant status and markers of oxidative stress in this population. Williams et al. [57] compared pre- and up to 12 months post-LTx levels of antioxidants (i.e., urate, ascorbate, thiols, α-tocopherol) and malondialdehyde (MDA, an end product of lipid peroxidation) in the blood and broncho-alveolar lavage (BAL) fluid of 19 recipients to those measured in 23 healthy controls. The poor antioxidant status seen in patients prior to LTx persisted during the first year after transplantation with little improvements. The increased concentration of MDA in both BAL fluid and serum in lung recipients indicate that these patients were oxidatively stressed, which may in turn explain the sustained depletion of antioxidant molecules. Events occurring in the early post-transplant period such as ischemia-reperfusion injury, infections and acute rejection are important sources of reactive oxygen species, which when produced in excess alongside with low antioxidant levels, leads to or enhances a pre-existing oxidative stress. Since ischemia-reperfusion injury, infections and acute rejection are all strongly associated with the development of BOS, one may speculate that oxidative stress is involved in the development and progression of this complication [58]. When the antioxidant status of serum and BAL fluid of lung recipients with and without acute rejection were compared, no difference was detected. However, following a longer post-transplantation time (median: 26 months), BAL fluid levels of antioxidants were found to be lower and oxidized glutathione (GSSG; a marker of oxidative stress) higher, albeit not significantly, in BOS individuals. These results suggest that depletion of antioxidant molecules is likely a consequence of the enhanced neutrophil infiltration and activation seen in BOS patients, which is hypothesized to generate large amounts of reactive oxygen species [59]. Despite some in vitro demonstration of the benefits of antioxidant molecules in attenuating inflammatory response of alveolar macrophages isolated from LTx patients [60], no randomized controlled trials on antioxidant supplementation have been carried out in this population. Whether these patients could potentially benefit from such an intervention remains to be determined.

### 2.4. Fluids and Electrolytes

Fluid and electrolyte management is a critical component of the early post-operative care. Microvascular leak in response to restoration of blood flow to the lungs following implantation leads to volume overload and pulmonary edema as well as hypotension. To reduce reperfusion edema and maintain hemodynamic stability, standard practice includes fluid restriction and the use of diuretics, which in turn, causes electrolyte imbalance especially of potassium, phosphorus and magnesium. Either low or high serum potassium levels were found in 69% of lung recipients placed on parenteral nutrition shortly after transplant [26]. Among contributing factors to hypokalemia are initiation of nutritional support, which enhances potassium movement into the cells, as well as the use of potassium-wasting diuretics. Hyperkalemia is postulated to be secondary to a decrease in renal function and the use of the immunosuppressant, cyclosporin A. Similarly, abnormalities in serum phosphorus were observed in 87% of recipients and were attributed to initiation of parenteral feeding causing hypophosphatemia and decreased renal function and excessive phosphate supplementation leading to hyperphosphatemia [26]. As for magnesium, normal concentration has been reported at transplant [61] but hypo- and hypermagnesemia were shown to occur in respectively 70 and 25% of lung recipients post-operatively [26]. Since the cyclosporine A-associated nephrotoxicity is postulated to cause hypomagnesemia through renal wasting, study participants received magnesium supplements as part of their post-transplant therapy [26,61]. Despite magnesium supplementation, serum magnesium declined in 37% of patients during the observation period (up to 500 days post-transplant) [61]. While reasons underlying this resistance to the supplementation effects are unclear, this study raises the importance of a regular monitoring of serum magnesium in LTx recipients, especially given that hypomagnesemia has been postulated to aggravate renal impairment and mediate the neurotoxic effects associated with cyclosporine A therapy [62]. Post-LTx hypomagnesemia is not only connected to the use of cyclosporine A but also of tacrolimus, the other calcineurin inhibitor, which is associated with nephrotoxicity [63]. Calcineurin inhibitors are believed to inhibit the expression of the magnesium transporter, transient receptor potential melastatin 6 (TRPM6), involved in renal magnesium reabsorption [64]. While tacrolimus-induced hypomagnesemia has been reported in all other solid organ transplants [65,66,67], it has not been specifically reported in the LTx context. Hypermagnesemia is less common and believed to result from a decline in renal function [26].

## 3. Nutritional Recommendations

Given the lack of clinical practice guidelines for nutrition management of LTx recipients, we tentatively formulated general recommendations based on the existing literature, clinical guidelines developed for other solid-organ transplant and non-transplant patients and current practice in our institution. These recommendations (Table 1) should not be considered definitive but clearly emphasize that more research is needed in this area. In addition, specific medical conditions are discussed in the subsequent section.

Nutrition management of LTx recipients should include a global nutritional assessment, an approach based on the practitioner’s clinical judgment. Furthermore, it requires a good knowledge of the underlying disease, how post-transplant phases influence nutritional requirements and the factors affecting calorie, macro- and micronutrient requirements. In the postoperative and short term period following transplant, nutritional goals involve the supply of adequate nutrients to: (1) correct or prevent nutritional deficiencies, (2) support the healing process for anastomosis and surgical wounds as well as the high nutritional demands present in the early postoperative period, and (3) maintain and optimize nutrient stores to strengthen the immune system to effectively fight infections and facilitate participation to rehabilitation and the return to normal daily activities. As time from transplant increases, nutritional goals change to rather focus on the prevention and management of complications associated with LTx such as obesity, hypertension, dyslipidemia, NODAT, osteoporosis, and renal insufficiency [68,69]. 

Nutrition management of LTx recipients also encompasses interventions and counseling regarding drug-nutrient interactions, safe food handling to prevent foodborne illnesses and gastrointestinal complications (e.g., gastroesophageal reflux disease (GERD), dysphagia, gastroparesis, bezoar, ileus, distal intestinal obstruction syndrome (DIOS), diarrhea, and constipation). While such interventions are an integral part of the post-LTx nutrition care, they will not be covered in this review due to scope limitation. 

### 3.1. Energy and Macronutrients

After surgery and in the absence of any complication, recipients generally remain in the ICU for a period of 1–3 days. Prolonged mechanical respiratory support is associated with longer ICU stay. During the early postoperative period, patients are in a high catabolic state where presence of the acute inflammatory process may limit response to aggressive nutrient delivery [70]. Overfeeding should be avoided as it can compromise weaning from mechanical ventilation [71]. No studies have specifically addressed nutritional needs of LTx recipients during the ICU stay and its relationship with outcomes such as mortality, ventilator-free days, infections or length of stay in the ICU although some evidence suggests that cumulative calorie and protein deficits during the ICU stay are associated with worse outcomes [72]. In practice, the recently updated nutrition guidelines for critically ill adults may apply to the LTx population [73]. When indirect calorimetry is unavailable to objectively quantify energy needs, total daily energy targets of 25–30 kcal/kg may represent a good starting point as well as other published predictive equations [73,74]. However, caloric requirements should be customized according to pre-transplant nutritional status, medical complications (e.g., sepsis, rejection) and particular conditions such as maldigestion and malabsorption in CF recipients. For example, an underweight pancreatic insufficient CF recipient with sepsis may require as high as 40–50 kcal/kg/day. Since little research has been undertaken in lung recipients to quantify energy requirements, total daily calorie recommendations for LTx recipients in the post-operative period are extrapolated from data generated in other solid organ transplants and in the general surgical population where energy requirements are estimated to be 1.35 to 1.75 times the REE [2], which corresponds roughly to 30–35 kcal/kg. After resolution of the stress response, the early catabolic phase progressively switches to an anabolic recovery phase characterized by the replenishment of the body’s depleted stores and weight gain, which necessitates a periodic reassessment of calorie requirements. Over the long term, the rate of weight gain slows and weight stabilizes to a healthy weight [9]; however, in some patients, excessive weight gain takes place and weight management, including a physical activity program and nutrition counselling, should be initiated to prevent the development of obesity and concomitant comorbidities. Discussions regarding acceptance of body image changes and weight gain are also part of long-term nutrition management. 

In the immediate phase after transplant, protein catabolism is markedly increased, and nutrition therapy should focus to minimize losses and preserve current lean body mass. As such, patients should receive 1.3 to 1.5 g of protein/kg body weight; however protein needs could increase up to 2.5 g/kg in the presence of severe malnutrition, high steroid dose, leak from chest tube drainage, compromised wound healing, infections or rejection [2]. In addition to these estimates, nitrogen balance, when available, may help monitor improvements in protein status and assess whether protein provision should be further adjusted [73]. Serum protein markers, such as albumin, are not considered good indicators of protein and nutritional status and therefore should not be relied upon to assess protein requirements. As time from transplant increases, the protein recommended daily allowance (RDA) for adults (i.e., 0.8 g/kg) applies. However, this recommended intake does not take into consideration the chronic intake of corticosteroids, which, even when lower doses are used, may still induce protein catabolism, although this has never been specifically evaluated over the long term. Given the importance of proteins for the maintenance of lean body mass and optimal immune function and the deleterious impact of corticosteroids on protein metabolism, protein requirements of lung recipients should rather be closer to 1 g/kg [68]. 

Chronic kidney disease (CKD) is a comorbidity that develops in the long-term post-transplant period as a consequence of the immunosuppressant-inducing nephrotoxicity. Its prevalence at 5 years post-transplantation was reported to be 16% in LTx recipients [75]. A protein restricted-diet is often recommended in non-transplant CKD management to reduce the kidneys’ workload. Nutritional status of LTx patients should be carefully assessed with regard to corticosteroid doses, protein status and kidney function prior to applying a stricter control of protein intake.

When glucose metabolism is normal, intakes within the acceptable macronutrient distribution range (AMDR) for carbohydrates (i.e., 45 to 65% of total calories) are recommended [76]. If hyperglycemia occurs after transplantation, close blood glucose monitoring, and nutritional and medical therapy should be promptly initiated. In patients who develop NODAT, non-pharmacological management includes lifestyle intervention and dietary modifications adjusted to the patient’s nutritional status and calorie needs with the goal of achieving and maintaining a healthy body weight [77,78]. Macronutrient distribution range (% of total calories) should be aligned with those recommended for people with diabetes: carbohydrates: 45–60%, proteins: 15–20% and fat: 20–30% [77]. Nutritional counselling may also emphasize that added sucrose and fructose intake should be limited to a maximum of 10% of total calories. To facilitate the control of blood glucose levels, the preferential selection of foods with a low glycemic index is also advised [77].

If lipid profile remains within normal range, practice is to recommend fat intakes that fall within the AMDR for lipids (i.e., 20 to 35% of total calorie intake) [76]. If dyslipidemia develops over time, dietary interventions for the prevention and management of dyslipidemia in non-transplant individuals are advised [79]. Efforts are made to promote healthy eating, physical activity and lifestyle choices to achieve and maintain a healthy body weight. Healthy eating patterns such as the Mediterranean, Dietary Approaches to Stop Hypertension (DASH) or Portfolio diet, vegetarian diets or patterns characterized by large consumption of nuts, legumes, olive oil, fruits and vegetables with a high fiber content and a low-glycemic load are encouraged based on the preferences and values of individuals and with the goal of achieving long-term adherence. Trans fats should be avoided and replaced by saturated and polyunsaturated fats while limiting intake of saturated fats to less than 9% of total calories [79].

Finally, abstinence or very moderate and occasional alcohol consumption is advocated given the negative effects of ethanol on two organ systems adversely impacted by LTx-related medications, namely bone and liver, and the association between alcohol and the occurrence of malignancies in the general population [80]. 

### 3.2. Micronutrients

In the early postoperative period, factors such as enhanced inflammation secondary to the transplant procedure itself, oxidative stress and wound healing process are believed to increase the demand for nutrients actively involved in these processes such as vitamins A and C, and zinc; however, no experimental evidence supports this assumption in LTx recipients. In the absence of recommendations regarding the optimal dose for supplementation, intakes around the age- and sex-specific RDAs but below the upper level (UL), should be encouraged for most micronutrients. Decision to supplement depends on the global nutritional assessment and laboratory results to adjust doses and avoid hypo- or hypervitaminosis. 

The large doses of corticosteroids used in the early post-transplant exert a negative impact on the metabolism of vitamin D and calcium by increasing vitamin D turnover and reducing calcium absorption [81]. In addition, tacrolimus and cyclosporine A were shown to enhance urinary excretion of calcium [82]. Long term post LTx requirements for calcium and vitamin D are linked to the increased risk of osteoporosis induced by the lifelong immunosuppressive regimen. Bone protection management should include an optimal intake of 1200 mg/d of elemental calcium through diet and supplements combined with a daily supplementation of 1000 IU of vitamin D_3_ or more to achieve optimal vitamin D status and prevent osteoporosis [83]. Given the rapid loss of bone mass shortly after transplant, such supplementation regimen is generally initiated early after LTx before hospital discharge. Impaired vitamin D status is common in lung recipients and was shown to persist in 47% and 27% of individuals despite routine supplementation with doses ranging from 880 to 1000 IU daily to 50,000 IU once or twice weekly [38,39]. Although such a persistent poor status may reflect compliance issues, it may also indicate that lung recipients need more vitamin D to maintain an adequate status due to the presence of aggravating factors specific to this population. Vitamin D status should therefore be monitored closely and dosages adjusted accordingly to maintain serum 25OHD levels above 75 nmol/L (i.e., 30 ng/mL), a cut-off point associated with better outcomes in this population [38,39]. 

Benefits of calcium supplements have been the topic of considerable controversy in recent years with some studies, conducted primarily in elderly subjects, suggesting that their use was harmful to cardiovascular health [84,85]. Postulated biological mechanism underlying this association relates to an increased risk of vascular calcification secondary to the rise of serum calcium following supplement intake. However, the elevation of serum calcium experienced after supplementation is modest and unlikely to be large enough to influence soft tissue calcification, a phenomenon more likely attributed to local tissue factors [86]. Moreover, studies reporting the association between calcium supplements and adverse cardiovascular events have been questioned regarding the potential of bias and confounding. These studies have been designed in the first place to test the impact of calcium supplementation on BMD with adverse cardiovascular events being secondary outcomes. Risk factors for cardiovascular disease were therefore not taken into consideration in the randomization scheme, resulting in groups that may have been unbalanced for potential confounders. Results from ongoing randomized controlled trials on the effect of calcium supplementation on cardiovascular events, as primary endpoints will help to clarify this controversial topic.

### 3.3. Fluids and Electrolytes

To limit reperfusion edema and primary graft dysfunction, current strategies in post operative management suggest restricting fluid administration [87] including limiting dietary fluid intake to 1.2–1.5 L/day until removal of the thoracic drainage tube. After this period, abundant fluid intake is insisted on to ensure normal diuresis and preserve kidney function. 

A low-sodium diet is generally advocated to minimize sodium-water retention and edema as well as to prevent and help to control high blood pressure that occurs secondarily to the use of corticosteroids and immunosuppressive drugs. In the absence of evidence-based guidelines for lung recipients, it is encouraged to follow the health behaviour management recommended for the prevention and treatment of hypertension, which includes reduction of sodium intake to 2 g per day, adhesion to the DASH pattern, limitation of alcohol consumption, regular physical exercise and achievement and maintenance of a healthy body weight [88].

In the immediate post-transplantation phase, the diuretic-induced electrolyte loss requires a close monitoring especially of potassium and magnesium levels. Persistent hypomagnesemia is frequent and dietary intakes alone are generally insufficient to maintain normal blood levels. Depending on the laboratory test results, intravenous or oral magnesium replacement therapy may be initiated. Calcineurin inhibitors are known to induce hyperkalemia. To maintain levels in the normal range and prevent cardiac arrhythmia, a low-potassium diet, with a moderate restriction of 1 mmol/kg or 60 mmol/day, may be necessary. Over time, dietary modifications may be required for the management of CKD with individualized electrolyte restriction based on blood analysis and nutritional status.

## 4. Special Considerations 

In addition to the aforementioned challenges and considerations associated with post-transplant nutrition management, there are situations requiring specific adjustments of nutrition care. Some of these conditions are detailed in this section.

### 4.1. Specialized Nutritional Support 

In the absence of complication, weaning from mechanical ventilation is usually performed 24 to 48 h post-LTx after which the recipient could start eating by mouth and progress as tolerated. The decision to initiate specialized nutritional support in the early post-operative period should be made in accordance with the global nutritional assessment, which takes into account the patient’s overall condition, nutritional status, and laboratory results, guided by clinical guidelines in non-transplant populations [73] and based on center experiences. When patients are unable to meet their oral nutrient requirements before transplant, enteral nutritional support by percutaneous endoscopic gastrostomy (PEG) or percutaneous endoscopic jejunostomy (PEG-J) could be initiated prior to transplantation and the feeding tube used early after LTx if enteral nutritional support is indicated. Specialized nutritional support is not static, but requires ongoing assessment and reevaluation and the decision to begin, continue or cease should be made following a comprehensive nutritional assessment. 

### 4.2. Cystic Fibrosis

Nutrition management is paramount to the care of CF patients [89] and remains equally important after LTx given the persistence of pancreatic insufficiency, malabsorption and the risk of nutritional deficiencies. Pre-transplant malnutrition, common in these patients, could be promptly reversed after LTx due to a combination of improved appetite and reduced energy requirements associated with decreased inflammation and work of breathing, and lower occurrence of respiratory infections. Consequently, energy requirements should be assessed on a regular basis and calorie intakes adjusted to achieve and maintain a healthy BMI and prevent excess weight gain. Overweight and obesity are an emerging concern in the non-transplant CF population [90,91] whereas data are scarce for CF recipients. Of note, CF patients have been encouraged to eat unrestricted high-energy, high-fat diets throughout their life course. It could be challenging to correct these lifelong eating habits as such permissive diet may no longer be required after transplant. Energy needs and macronutrient requirements should therefore be regularly reassessed in these patients according to the correction of malnutrition, weight evolution and co-morbidities related to LTx. In non-transplant CF patients, protein digestive capacity is impaired but normalized with intake of pancreatic enzymes [92]. It is recommended that protein intake should constitute at least 20% of daily calorie intake [89]. While no data exists on protein requirements of CF recipients, the same recommendation should be extended to the post-transplant period. In terms of micronutrients, CF recommendations for vitamin D and K continue to apply [89,93]; however nutritional status of vitamin A and E should be closely monitored as serum levels of these vitamins may increase after transplant [50]. Serum vitamin A and E must be checked 3 to 6 months post-transplant and annually thereafter. If blood levels rise, supplement doses of vitamin A and E should be reduced or discontinued. Individuals with CF lose more sodium chloride in their sweat than non-CF individuals and, to avoid salt depletion, they are instructed to eat liberal amounts of dietary sodium especially in conditions associated with increased sweating (e.g., fever, exercise, hot weather) [94]. As no study has yet addressed sodium needs of individuals with CF after LTx, there is little evidence to support a change in recommendations prevailing in non-transplant CF patients. However, one should question whether such large intakes of sodium in the post-transplant period might contribute to the risk of developing or exacerbating kidney disease or liver dysfunction over the long term. 

Individuals with CF are more at risk of developing comorbidities such as diabetes [95] and bone [96] and liver disease [97] prior to transplant. The risk of developing or aggravating such comorbidities is amplified in the post-transplant period given the negative impact of the immunosuppressive therapy on many organ systems. Post-transplant screening should be actively pursued and nutrition management of CF-related comorbidities initiated when necessary [30,78,95,96,97].

### 4.3. Sarcoidosis 

Sarcoidosis is a granulomatous disease that may progress to pulmonary fibrosis necessitating LTx. Macrophages within sarcoid granulomas produce calcitriol, the bioactive form of vitamin D, which in turn may result in hypercalcemia and hypercalciuria. Prevalences range from 11% for hypercalcemia to 40% of sarcoidosis patients for hypercalciuria [98]. Given that calcium and vitamin D supplements are widely recommended for post-transplant osteoporosis prophylaxis, it is unclear whether such supplements should be safely given to these patients. In their retrospective study of 301 sarcoidosis patients, Kamphuis et al. found that 5% of individuals supplemented with calcium and vitamin D developed hypercalcemia compared to 11% of unsupplemented patients [99]. In addition, the authors reported that 63% of patients had insufficient serum 25OHD levels (<50 nmol/L or 20 ng/mL), and that serum 25OHD was inversely related to disease activity of sarcoidosis. Although the experimental design precludes any causal inferences, the authors concluded that, rather than being detrimental, vitamin D supplementation may benefit individuals with sarcoidosis [99]. 

### 4.4. Chylothorax

Chylothorax is a well-known complication of thoracic surgery characterized by the accumulation of chyle in the pleural space, usually consequent to the disruption or obstruction of the thoracic duct and leakage of lymphatic vessels. As a result, triglyceride-rich chylomicrons leak into the pleural cavity and the presence of chylomicrons or concentration of triglycerides >1.24 mmol/L (110 mg/dL) in pleural effusions are considered diagnostic criteria. For yet unclear reasons, this complication is particularly prevalent in patients with lymphangioleiomyomatosis (LAM) both before and after LTx [100]. There is a lack of consensus regarding the optimal management of chylothorax and treatment options range from nutritional interventions to surgical and non-surgical procedures [101,102]. Nutritional therapy is part of the conservative and usually first-line treatment, which primarily consists in reducing chyle production and flow to allow spontaneous closure of the chyle leak. Interventions include a very low fat diet (i.e., <10 g/day) to limit fat absorption through the lymph vessels and thoracic duct. Given the very restrictive nature of this diet, this intervention should not last more than 2 weeks [103]. The very low-fat diet should be combined with supplemental medium-chain triglyceride (MCT) oil, which bypasses typical lipid absorption routes and is absorbed directly into the portal vein. MCT oil may be mixed with foods and dosage increased up to 4 to 5 tablespoons a day according to digestive tolerance. However, MCT may make up part of the lymph fluid as it has been reported, in individuals provided an enteral formula with MCT as the sole fat source, that chyle fluid still had appreciable amounts of MCT [104]. As chyle contains considerable amounts of proteins, lipids, fat-soluble vitamins, fluids and electrolytes, patients with chylothorax may experience a rapid nutritional decline that adds to the morbidity. Therefore, a high protein diet supplemented with fat-soluble vitamins and essential fatty acids (i.e., linoleic acid and α-linolenic acid) should be initiated along with the dietary fat modifications [105]. In addition, fluid and serum electrolytes should be monitored closely to prevent depletion. Enteral nutritional feeding with elemental formulae is recommended if patients are unable to adhere to the very low fat diet while total parental nutrition is indicated in patients where other options have failed or who need additional nutrition support to meet their nutrient requirements.

## 5. Conclusions

LTx recipients represent a population of patients with a broad spectrum of nutrient requirements, which are affected by a host of factors ranging from the post-transplant stage to manifestations of the underlying disease, the medication and doses used and individual characteristics. Although nutrition is crucial for the short- and long-term success of LTx, more research is needed to better characterize post-transplant nutrient requirements and test the efficacy of nutrition interventions. Such research findings will provide evidence for the development of clinical guidelines for nutritional management of lung transplant recipients, which are currently lacking. Meanwhile, a comprehensive nutrition assessment combined with a good knowledge of nutritional issues and challenges and ways to address them will help lung recipients to live a longer and healthier life.

## Figures and Tables

**Table 1:** Summary of nutritional requirements for lung transplant recipients.

Nutrients	Acute Post-Transplant Phase	Chronic Post-Transplant Phase
Energy	Indirect calorimetry (if available) 25–35 kcal/kg	Adjust to achieve and maintain healthy body weight
Protein	1.3–2.5 g/kg	1–1.2 g/kg (adjusted to corticosteroid doses)
Carbohydrates	Limit simple carbs if hyperglycemic	45–65% of total calories NODAT: 45–60% of total calories Added sugars: maximum 10% of total calories Focus on foods with low glycemic index
Fat	No specific recommendation	20–35% of total calories If dyslipidemia: healthy dietary patterns (e.g., Mediterranean diet) Saturated fats: <9% of total calories
Fluids	Restricted to maintain hemodynamic stability	Liberal/Abundant
Sodium	Low-sodium diet	If high blood pressure: low-sodium & DASH diet
Magnesium	Supplement often required	Supplement often required
Potassium	Supplement often required Unrestricted unless necessary	Unrestricted unless necessary
Iron	Age and sex-specific RDA Supplement based on bloodwork	Age and sex-specific RDA Supplement based on blood work
Zinc	Age and sex-specific RDA Supplementation may be necessary in the context of increased demands (e.g., dehiscence, delayed wound healing)	Age and sex-specific RDA
Calcium	1200 mg/day from diet + supplement	1200 mg/day from diet + supplement
Vitamin D	Daily 1000 UI or more based on bloodwork	Daily 1000 UI or more based on blood work
Vitamin A	Age and sex-specific RDA Same dosage regimen as pre-LTx Supplementation may be necessary in the context of increased demands (e.g., dehiscence, delayed wound healing)	Age and sex-specific RDA Adjust supplement dosage based on blood work CF recipients: May need to stop supplementation
Vitamin E	Age and sex-specific RDA Same dosage regimen as pre-LTx	Age and sex-specific RDA Adjust supplement dosage based on blood work CF recipients: May need to stop supplementation
Vitamin C	Age and sex-specific RDA Supplementation may be necessary in the context of increased demands (e.g., dehiscence, delayed wound healing)	Age and sex-specific RDA
B vitamins	Age and sex-specific RDA Supplement based on blood work	Age and sex-specific RDA Supplement based on blood work
Vitamin K	Age and sex-specific AI CF recipients: 1–10 mg/day	Age and sex-specific AI CF recipients: 1–10 mg/day

Use dry and/or adjusted body weight. LTx: lung transplant; NODAT: New onset diabetes after transplantation; CF: cystic fibrosis; RDA: recommended dietary allowance; AI: adequate intake.
